# Delayed ventilation assessment using fast dynamic hyperpolarised Xenon-129 magnetic resonance imaging

**DOI:** 10.1007/s00330-019-06415-1

**Published:** 2019-09-04

**Authors:** Mitchell Chen, Ozkan Doganay, Tahreema Matin, Anthony McIntyre, Najib Rahman, Daniel Bulte, Fergus Gleeson

**Affiliations:** 1 The Churchill Hospital, Oxford University Hospitals NHS Foundation Trust, Old Road, Oxford, OX3 7LE UK; 2 Department of Oncology, Oxford University, Old Road Campus Research Building, Roosevelt Drive, Oxford, OX3 7DQ UK; 3 Oxford NIHR Biomedical Research Centre, The Churchill Hospital, Old Road, Oxford, OX3 7LE UK; 4 The Institute of Biomedical Engineering, Oxford University, Old Road Campus Research Building, Roosevelt Drive, Oxford, OX3 7DQ UK

**Keywords:** Chronic obstructive pulmonary disease, Lung, Magnetic resonance imaging, Xenon

## Abstract

**Objectives:**

To investigate the use of a fast dynamic hyperpolarised ^129^Xe ventilation magnetic resonance imaging (DXeV-MRI) method for detecting and quantifying delayed ventilation in patients with chronic obstructive pulmonary disease (COPD).

**Methods:**

Three male participants (age range 31–43) with healthy lungs and 15 patients (M/F = 12:3, age range = 48–73) with COPD (stages II–IV) underwent spirometry tests, quantitative chest computed tomography (QCT), and DXeV-MRI at 1.5-Tesla. Regional delayed ventilation was captured by measuring the temporal signal change in each lung region of interest (ROI) in comparison to that in the trachea. In addition to its qualitative assessment through visual inspection by a clinical radiologist, delayed ventilation was quantitatively captured by calculating a covariance measurement of the lung ROI and trachea signals, and quantified using both the time delay, and the difference between the integrated areas covered by the signal-time curves of the two signals.

**Results:**

Regional temporal ventilation, consistent with the expected physiological changes across a free breathing cycle, was demonstrated with DXeV-MRI in all patients. Delayed ventilation was observed in 13 of the 15 COPD patients and involved variable lung ROIs. This was in contrast to the control group, where no delayed ventilation was demonstrated (*p* = 0.0173).

**Conclusions:**

DXeV-MRI offers a non-invasive way of detecting and quantifying delayed ventilation in patients with COPD, and provides physiological information on regional pulmonary function during a full breathing cycle.

**Key Points:**

*• Dynamic xenon MRI allows for the non-invasive detection and measurement of delayed ventilation in COPD patients.*

*• Dynamic xenon MRI during a free breathing cycle can provide unique information about pulmonary physiology and pulmonary disease pathophysiology.*

*• With further validation, dynamic xenon MRI could offer a non-invasive way of measuring collateral ventilation which can then be used to guide lung volume reduction therapy (LVRT) for certain COPD patients.*

**Electronic supplementary material:**

The online version of this article (10.1007/s00330-019-06415-1) contains supplementary material, which is available to authorized users.

## Introduction

First described by Van Allen et al [1] in 1931, pulmonary collateral ventilation refers to the phenomenon of ventilation of alveolar structures through passages or channels other than the normal airways [2]. It is known to be associated with emphysema in patients with chronic obstructive pulmonary disease (COPD) where it is believed that pathological distal airspace dilatation and wall destruction lead to the formation of alternative channels of airflow [3]. However, no clear relationship has been established between the presence and extent of collateral ventilation and COPD disease severity [3].

In order to determine the likelihood of treatment success when considering patients for treatment options targeting pulmonary hyper-expansion in severe COPD, such as endobronchial valve placement or surgical lung resection, the assessment of collateral ventilation is essential [4]. In current clinical practice, COPD patients can be assessed for the presence of collateral ventilation either indirectly with computed tomography (CT) or directly via a commercially available bronchoscopic system (Chartis® system, Pulmonx Inc.) [5, 6]. There is a need for alternative non-invasive functional imaging methods to assess patient lungs for collateral ventilation, because bronchoscopic systems are invasive, and chest CT provides limited functional pulmonary information and involves ionising radiation.

^133^Xe scintigraphy [7] and xenon-enhanced dynamic dual-energy CT [8, 9] have previously been employed to obtain images of collateral ventilation but incur exposure to ionising radiation. Hyperpolarised gas imaging has emerged as a valuable technique for evaluating ventilation [10–12], offering the advantage of enabling the assessment of gas diffusion to comprehensively evaluate both emphysema distribution and collateral ventilation [13]. Hyperpolarised ^3^He MRI has successfully demonstrated collateral ventilation directly [14], and long-range diffusion measurements with ^3^He may indicate that collateral ventilation is taking place [14].

Hyperpolarised ^3^He MRI has been more extensively studied than ^129^Xe MRI due to its stronger magnetic moment, resulting in a higher spatial resolution and signal-to-noise ratio than ^129^Xe [15]. However, the use of ^129^Xe is gaining popularity in recent years due to its abundant natural supply and lower cost [16]. Recent breakthroughs in ^129^Xe production have allowed it to achieve levels of polarisation similar to those with ^3^He [17]. Positive correlations have been established between functional ventilation information acquired using hyperpolarised ^3^He and hyperpolarised ^129^Xe MRI [18]. The higher density of ^129^Xe makes it more sensitive to capturing ventilation defects [19]. Its greater solubility and larger chemical shift enable the acquisition of additional functional information about gas exchange [20–22]. Both gases, at the imaging doses prescribed, are well-tolerated by both healthy individuals and patients with lung diseases [23–25]. More recently, hyperpolarised ^129^Xe has been shown to be a promising tool for measuring lobar ventilation [26, 27], and for identifying temporal changes in the arrival of xenon gas to different parts of the lung [28, 29].

Most existing studies [14, 30] on the use of hyperpolarised gas MRI for characterising delayed ventilation in COPD patients are based on time-resolved static MR sequences during a single breath-hold. However, relatively slowly filling ventilation defects can be challenging to assess in one breath-hold. Multi-breath imaging techniques have been proposed and tested [31, 32], where during each breath, a fraction of the hyperpolarised gas is replaced by newly arrived gas, and as new scans are acquired at each subsequent breath-hold, a volume fractional ventilation measure is calculated and used to assess air trapping and delayed ventilation. However, these methods are susceptible to lung movements, which give rise to localisation errors, as well as signal decays due to gas relaxation between scan acquisitions.

The static nature of the aforementioned methods makes them incapable of capturing the dynamics of pulmonary function during different phases of a breathing cycle, which can lead to a failure in identifying lung areas with significant ventilation problems, such as the ones affected by collateral ventilation and pulmonary air leaks. An understanding of the dynamic ventilation process during a complete breathing cycle could offer unique insight into pulmonary physiology and pathophysiology in disease states. Finally, the length of breath-hold required for high quality scans in static hyperpolarised gas MRI often proves challenging for patients with severely diseased lungs.

In addition to an earlier ^3^He-based work [33] supporting the idea of dynamic ventilation MRI during free breathing, two more recent pilot studies [34, 35] using dynamic ^129^Xe MRI have reported promising physiologically compatible results in gas phantoms, healthy volunteers and a limited number of COPD patients, prompting further research into this topic. In this paper, a fast dynamic hyperpolarised ^129^Xe ventilation magnetic resonance imaging (DXeV-MRI) method is presented, for assessing delayed ventilation in patients with COPD.

## Methods and materials

The study was approved by the UK National Research Ethics Service South Central Committee (Berkshire UK, REC 11/SC/0487 and REC 11/SC/0488). Written informed consent was obtained from all study participants.

Three male participants (aged 31, 34 and 43, respectively) with healthy lungs were imaged between September 2017 and May 2018. Fifteen patients (M/F = 12:3, age range 48–73) diagnosed with COPD were prospectively enrolled from a tertiary referral centre and imaged between February 2016 and August 2017. The study recruitment criteria are given in the appendices.

Participants in both cohorts underwent a spirometry tests, DXeV-MRI at 1.5-Tesla and quantitative computed tomography (QCT) of the chest at a single time point. In the COPD cohort, study measures were completed during disease stability, defined as following a 2-week window without COPD exacerbation or change in medications.

A summary of demographics for the patients with COPD included in the study is given in Table 1.

**Table 1:** Summary of our COPD cohort

Participant no.	Gender (M/F)	Age (years)	GOLD stage	FEV1 (% predicted)	FEV1/FVC	%LAA
1	M	70	II	68.3	53.9	24.3
2	M	63	II	58.1	44.4	10.8
3	M	59	IV	16.4	39.4	21.9
4	M	68	III	46.4	37.9	15.2
5	M	71	IV	18.6	32.2	6.2
6	F	58	II	57.2	54.0	0.3
7	M	73	III	24.7	28.7	3.7
8	M	67	III	40.6	37.3	36.2
9	M	58	III	46.9	45.2	11.4
10	M	64	III	49.0	61.1	23.9
11	M	72	II	61.4	69.1	0.3
12	M	72	IV	23.9	32.4	31.6
13	M	65	IV	26.3	35.0	15.8
14	F	68	IV	29.7	37.7	32.1
15	F	48	II	73.9	67.8	6.6

### ^129^Xe polarisation and delivery

Isotropically enriched ^129^Xe gas (86% ^129^Xe, Spectra Gases Inc.) was polarised to 10–15% by rubidium vapour spin-exchange optical pumping (SEOP), and cryogenically accumulated in 1-L doses using a commercial polariser (Model 9300, Polarean). Polarisation was measured using a commercial polarisation measurement station (Model 2881, Polarean).

Hyperpolarised ^129^Xe was thawed into a Tedlar® bag (Jensen Inert Products) and administered within 10 minutes of production to participants who were lying supine in the MRI scanner. Participants were given standard breathing instructions prior to image acquisition and asked to perform practice breaths. They are instructed to exhale to functional residual capacity (FRC) and then inhale the full 1-L gas content within 3 seconds during the inhalation period, followed by a 5-second breath-hold, then exhale the gas over 3 seconds. The total scanning time was 20 seconds, including a baseline period of 2 seconds before the initial inhalation and a flush interval of 5 seconds after the end of exhalation. Flush interval is the time interval between the exhalation of xenon gas and end of the scan, when the signal intensity drops significantly due to the reduced concentration of ^129^Xe and incoming oxygen.

### ^1^H-MRI

Both ^1^H-MRI and DXeV were performed on a 1.5-Tesla Signa HDx whole-body MR system (GE Healthcare). For ^1^H-MRI, plane localiser images were acquired using double-inversion-recovery black-blood imaging sequence with the following scanning parameters: number of slices: 13, slice thickness: 15 mm, imaging plane: coronal, and image reconstruction size: 128 × 128 pixels.

### DXeV-MRI

For DXeV-MRI, participants were fitted with a flexible twin Helmholtz quadrature transmit-receive coil (Clinical MR Solutions) tuned to the ^129^Xe Larmor frequency for 1.5 Tesla (~ 17.7 MHz).

The scans were acquired using a two-interleaved spiral k-space sampling approach, due to its superior temporal resolution, which has been demonstrated previously [35]. The following pulse sequence parameters were used: number of slices: 13, slice thickness: 15 mm, imaging plane: coronal, and image reconstruction size: 128 × 128 pixels. A total of 32 volume images were acquired during the 20-second scanning time, with each volume image taking 625 milliseconds. The total table time for each HP ^129^Xe-MRI scan is about 5 minutes.

### Spirometry tests

Spirometry tests were completed on all participants using a Compact plus flowmeter pulmonary function testing station (Hypairm Medisoft Group) and included forced expiratory volume in 1 second (FEV1) and FEV1/forced vital capacity (FVC). Recordings were compared to predicted values from standard published data for the given patient population.

### Quantitative CT

QCT was performed on a 16-slice GE Discovery 670 scanner (GE Healthcare). Images were acquired with a 1.25-mm slice thickness during suspended tidal inspiration following inhalation of 1 L oxygen via a Tedlar® bag from FRC to ensure lung volumes were as similar to HP ^129^Xe-MRI as possible. All participants had received breath-hold training beforehand to ensure scan reproducibility.

### Image data analyses

**Fig. 1:**
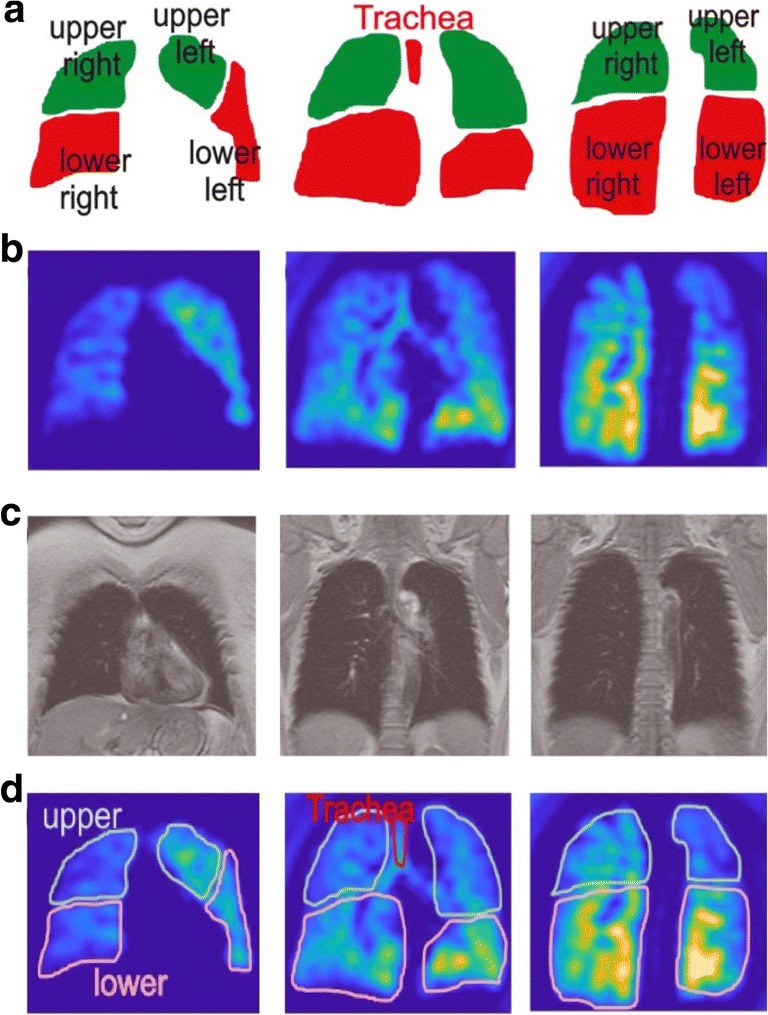
Representative regions of interest (ROIs), as manually specified on coronal slices; outlining the trachea, and upper and lower areas of each lung. **a** Region of interest mask contours. **b** Two interleaved high-temporal resolution DXeV-MRI images. **c** Corresponding ^1^H-MRI images. **d** User-defined tracheal and lung ROIs on DXeV-MRI images

Regions of interest (ROIs) were first manually delineated in the trachea, and the upper and lower parts of each lung by a clinical radiologist with 2 years of thoracic radiology experience, on every coronal slice (see Fig. 1a–d), based on the presence of oblique fissures on QCT and anatomical knowledge of the thoracic structures from the ^1^H-MRI scan. The lung ROIs were labelled as left upper, left lower, right upper and right lower lungs. Hyperpolarised ^129^Xe signal decay occurred to the flip angle and longitudinal decay of hyperpolarised ^129^Xe (T_1_ relaxation) during the acquisition of time-series images [35]. Decay of polarisation was accounted for through normalisation of images at each time point using a well-ventilated area in the trachea to address variations in scan conditions between subjects. To ensure consistency in normalisation, the final two timesteps (frames 31 and 32) were omitted in the analysis, because the tracheal signal is not always present in those frames for all study patients. All signals were also normalised to the maximum mean value measured during the breathing cycle, for the ease of comparison.

Pulmonary Toolkit (PTK), an open-source software, was used to analyse QCT. The percentage emphysema on a global and lobar basis was determined, in the latter case employing a fissure-based pulmonary lobar segmentation supported by software. The extent of emphysema was quantified as percentage area with an attenuation value of less than 950 Hounsfield Unit, given as median and 25th and 75th percentiles (%LAA).

Collateral ventilation manifests as delayed filling of ventilation defects on imaging. However, delayed filling of ventilation defects can also be attributed to partial obstruction, air trapping or narrowing of peripheral airways [14]. By definition, a signal can only be classified as collateral ventilation if the observed delayed ventilation originates from corresponding reduction in expected ventilation in an adjacent pulmonary airspace [36]. In this study, delayed ventilation was defined as a detectable increase or persistent high signal over baseline in a particular lung ROI that is not seen elsewhere in the ventilated lung. We would refer to any observed delayed filling as simply delayed ventilation and reserve the term collateral ventilation for when this delayed filling can be further validated using gold standard methods such as Chartis®.

For delayed ventilation detection, the covariance of signals was computed using the following equation (Eq. 1):

1 $$ \mathit{\operatorname{cov}}\ \left(X,Y\right)=\frac{\sum_{i=1}^n\left({x}_i-{\mu}_x\right)\left({y}_i-{\mu}_y\right)}{n-1} $$

where *X* and *Y* are the normalised temporal signals measured in the trachea and lung ROI, respectively; *x*_i_ and *y*_i_ are the signals in the trachea and lung ROI at timepoint *i*, respectively; *μ*_x_ and *μ*_y_ are their expected means; and *n* is the number of time steps during image acquisition. A value of 0.97 was chosen as the detection threshold, based on the calculated covariance of the control cohort, where visually there is minimal lobar collateral/delayed ventilation, in keeping with physiological expectation [36]. Cases with a covariance of less 0.97 were classed as having demonstrated delayed ventilation; all such cases were confirmed by visual inspection of the temporal signal maps by a clinical radiologist.

The amount of time delay was characterised by the temporal difference between the maximal ventilation signal in the trachea and that in the lung ROI. The tracheal ^129^Xe signal was normalised for each patient, since it is little affected by COPD and is most representative of true hyperpolarised MRI signal decay. In order for delayed ventilation to be observed, a minimum delay of 1 timestep (0.6 second) is required. This timescale is in keeping with that published in relevant literature [14].

**Fig. 2:**
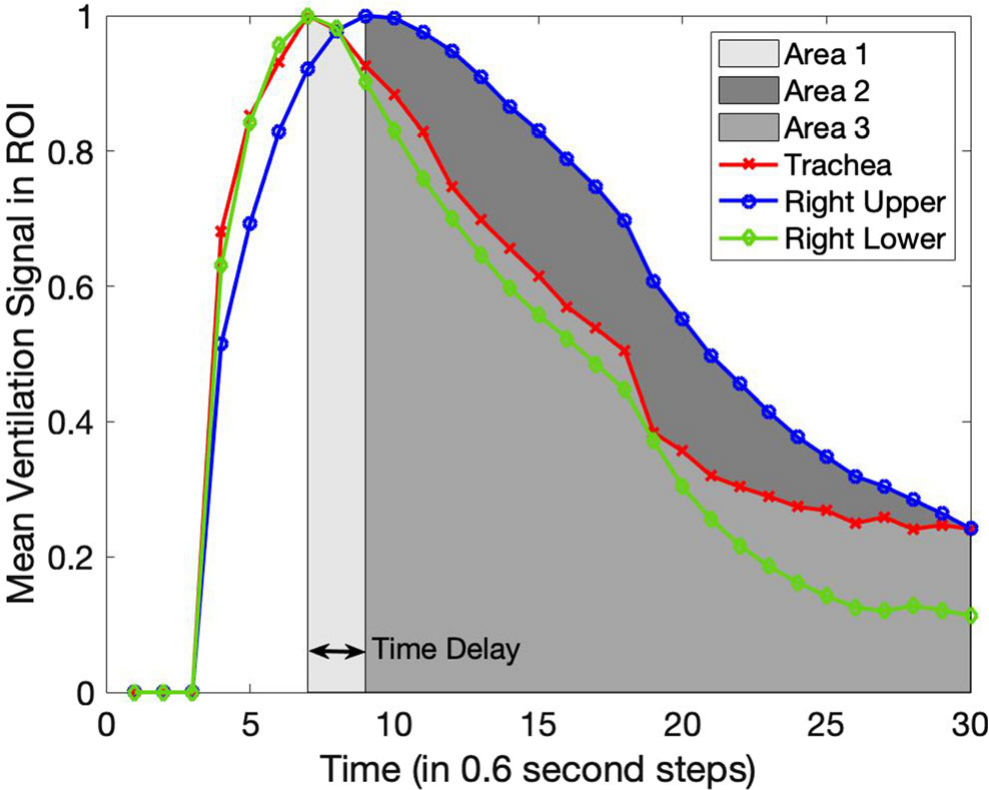
Illustration of the concepts of time delay and signal-time product difference (difference in the shaded areas, or Area 2 minus Area 1) in a case showing delayed ventilation. Area 3 represents the overlap of Areas 2 and 1

In cases where delayed ventilation was observed, it was quantified by computing the difference in the integrated areas of the signal-time curve of the trachea and that of the lung ROI in question, which is referred to simply as the signal-time product difference in this paper. This concept is illustrated in Fig. 2.

A clinical radiologist with 2 years of thoracic radiology experience reviewed the DXeV-MRI and corresponding QCT coronal images visually for delayed ventilation and any discernable structural differences, such as incomplete fissures, that might have contributed to it.

Image data processing and statistical analyses were performed using in-house software developed in a MATLAB® environment (MathWorks). A *p* value < 0.05 was considered to be statistically significant. Spearman’s coefficient was computed to assess the correlations of spirometry test measurements, %LAA, trachea-lung ROI covariance, time delay and the quantity of delayed ventilation (signal-time product difference).

## Results

**Fig. 3:**
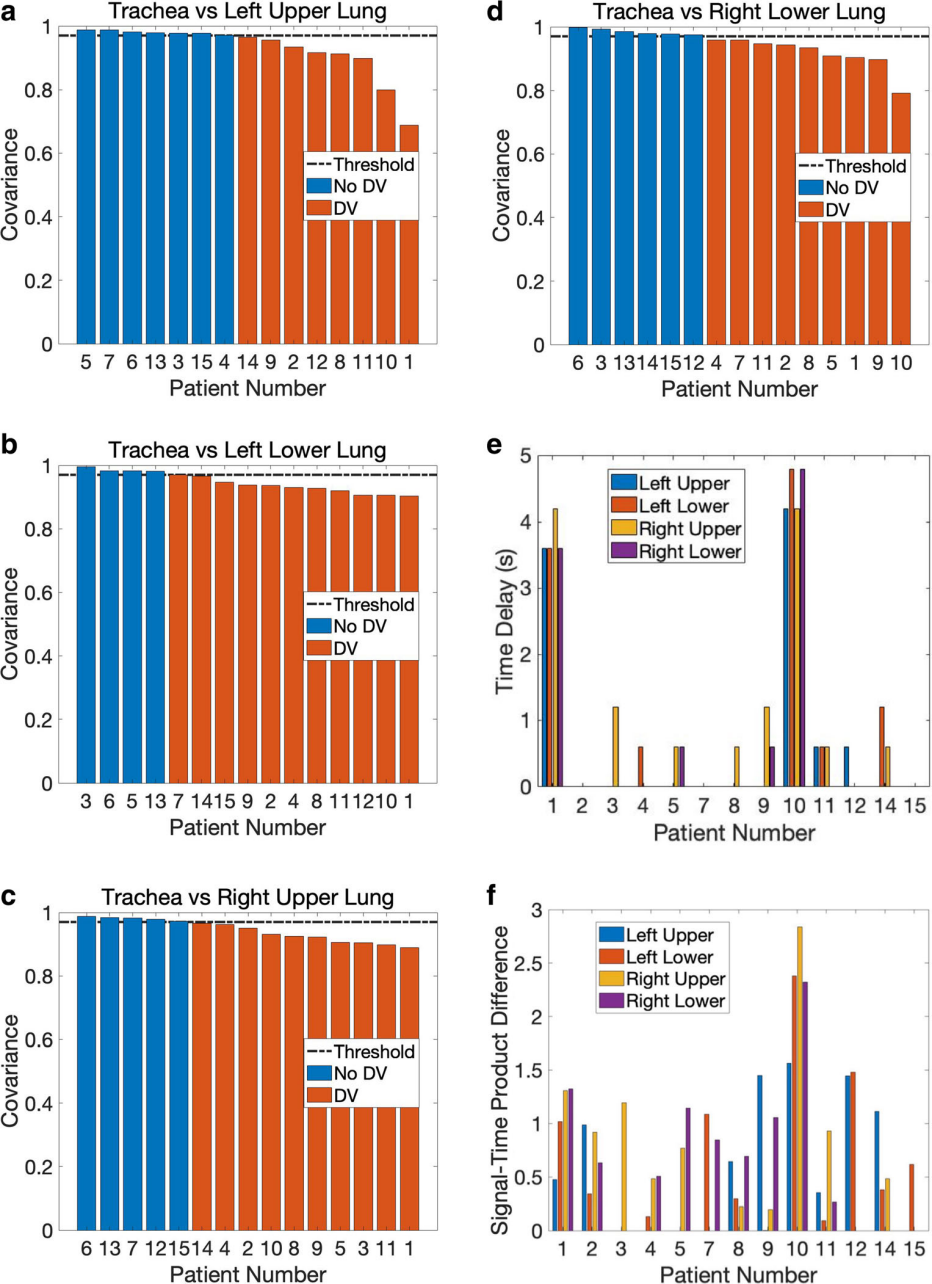
Co-variance analysis results for quantitative measurement of delayed ventilation. Delayed ventilation is considered detected when covariance < 0.97, and these cases are shown in red, otherwise in blue. **a** Trachea vs. left upper lung. **b** Trachea vs left lower lung. **c** Trachea vs. right upper lung. **d** Trachea vs right lower lung. **e** Time delay calculated based on the method shown in Fig. 2. Note that only those with at least one pulmonary ROI showing delayed ventilation are shown. **f** The quantity of delayed ventilation for patients where it is shown in at least one ROI, using the method shown in Fig. 2

To numerically capture and measure delayed ventilation, covariance was computed using Eq. 1. Results from the COPD cohort are given in Fig. 3a–d. Thirteen of the 15 COPD participants demonstrated some form of delayed ventilation, present in all four lung ROIs in six, three in two, two in three and one in the remaining two participants. By contrast, none of the participants in the control group demonstrated any delayed ventilation, both numerically and on visual inspection. The average covariance of the normal cohort is statistically significantly different from that of the COPD group (*p* = 0.0173).

For those where there was detectable delayed ventilation, the time delay and quantity of signal delay were calculated, the latter measured in signal-time product difference. The results are given in Fig. 3e and f, respectively. There were cases where a zero time delay was measured despite notable delayed ventilation; this is because of slower regional signal decay in the lung ROI in comparison to that in the trachea, rather than there being an actual temporal delay in its maximal signal.

Various correlation analysis results are given in Table 2. There was no statistically significant correlation with spirometry tests: between the covariance measure and %predicted FEV1 (*p* = 0.12), with delayed time (*p* = 0.84), nor quantity of delayed ventilation (*p* = 0.75). No correlation of statistical significance was found between %LAA and % predicted FEV1 (*p* = 0.57), which is in accordance with results reported in earlier literature [26]. However, statistically significant correlations between the trachea-lung ROI covariance and %LAA (*R* = − 0.40, *p* = 0.02) and time delayed (*R* = 0.34, *p* = 0.05) were established, both on a regional basis. No such correlation is, however, found with the quantity of delayed ventilation (*p* = 0.81).

**Table 2:** Correlation analysis results between QCT-derived %LAA, FEV1 and DXeV-derived delayed ventilation measurements. Spearman’s correlation coefficients are shown with *p* values in parentheses. *FEV1:* force expiratory volume in 1 second (% predicted), *%LAA:* emphysema score

Parameter	%LAA	Trachea-ROI covariance measurement	Time delay(s)	Quantity of delayed ventilation (signal-time product difference)
FEV1	− 0.15 (0.57)	− 0.41 (0.12)	0.06 (0.84)	− 0.09 (0.75)
%LAA	N/A	− 0.40 (0.02)	0.34 (0.05)	0.05 (0.81)

**Fig. 4:**
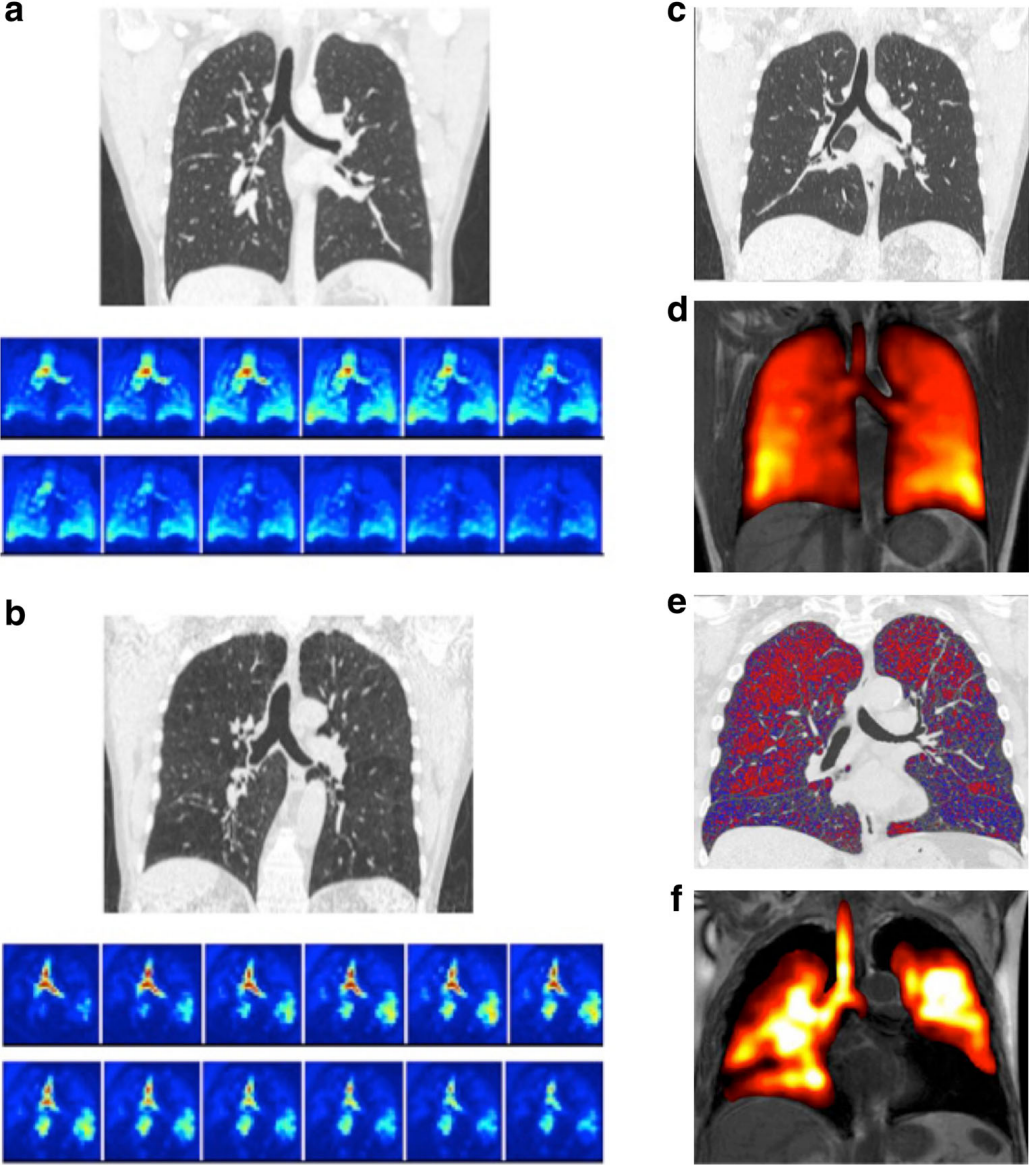
Dynamic pulmonary ventilation in (**a**) a healthy participant and (**b**) COPD Patient 1. The top and bottom subfigures in (**a**) and (**b**) show the QCT in lung window and temporal change in ventilation level during a natural breathing cycle, respectively. Note the marked ventilation defects demonstrated in the COPD patient. **c** CT of the participant in (**a**). **d** Fused DXeV-MRI and ^1^H-MRI for (**a**). **e** CT with highlighted emphysematous changes for the patient in (**b**). (**f**) Fused DXeV-MRI and ^1^H-MRI for patient in (**b**)

The CT and DXeV-MRI data from a control cohort participant with healthy lungs and a COPD patient (Patient 1) are given in Fig. 4a and b, respectively. The corresponding fused end-inspiratory DXeV-MRI and ^1^H-MRI for the same participants are presented in Fig. 4d and f, and the corresponding CT data are given in Fig. 4c and e, respectively. The healthy participant did not demonstrate any emphysematous change on CT (Fig. 4c), nor any ventilation defects on DXeV-MRI (Fig. 4d). The %LAA for the COPD patient, on the other hand, was measured to be 21.9% and 26.3% for left and right lungs, respectively, and is confirmed by the presence of ventilation defects in those lungs (Fig. 4f). For this patient, the covariance measurements of the left upper, left lower, right upper and right lower lungs compared to the trachea are 0.69, 0.88, 0.85 and 0.88, respectively. Their corresponding time delays are 3.6, 3.6, 4.2 and 3.6 seconds, respectively, and the quantities of delayed ventilation (signal-time product difference) are 0.47, 1.02, 1.31 and 1.32, respectively.

**Fig. 5:**
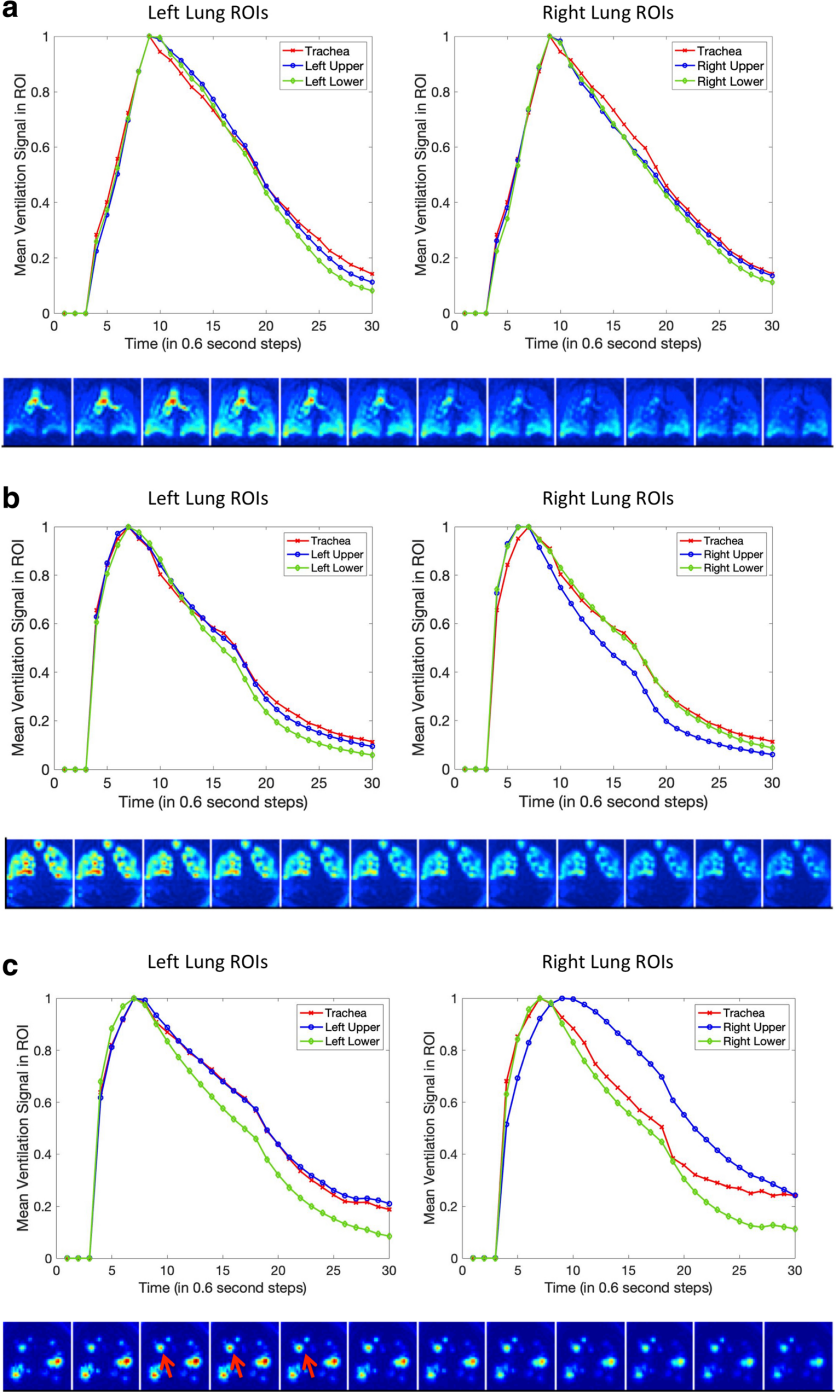
Normalised temporal mean ventilation in the trachea and lung ROIs and DVeX-MRI images for (**a**) a control cohort participant and (**b**) COPD Patient 6, with no detectable delayed ventilation, and (**c**) COPD Patient 3, with delayed ventilation in the right upper lobe (red arrows). The DVeX-MRI image sequences are truncated to highlight the key features

**Fig. 6:**
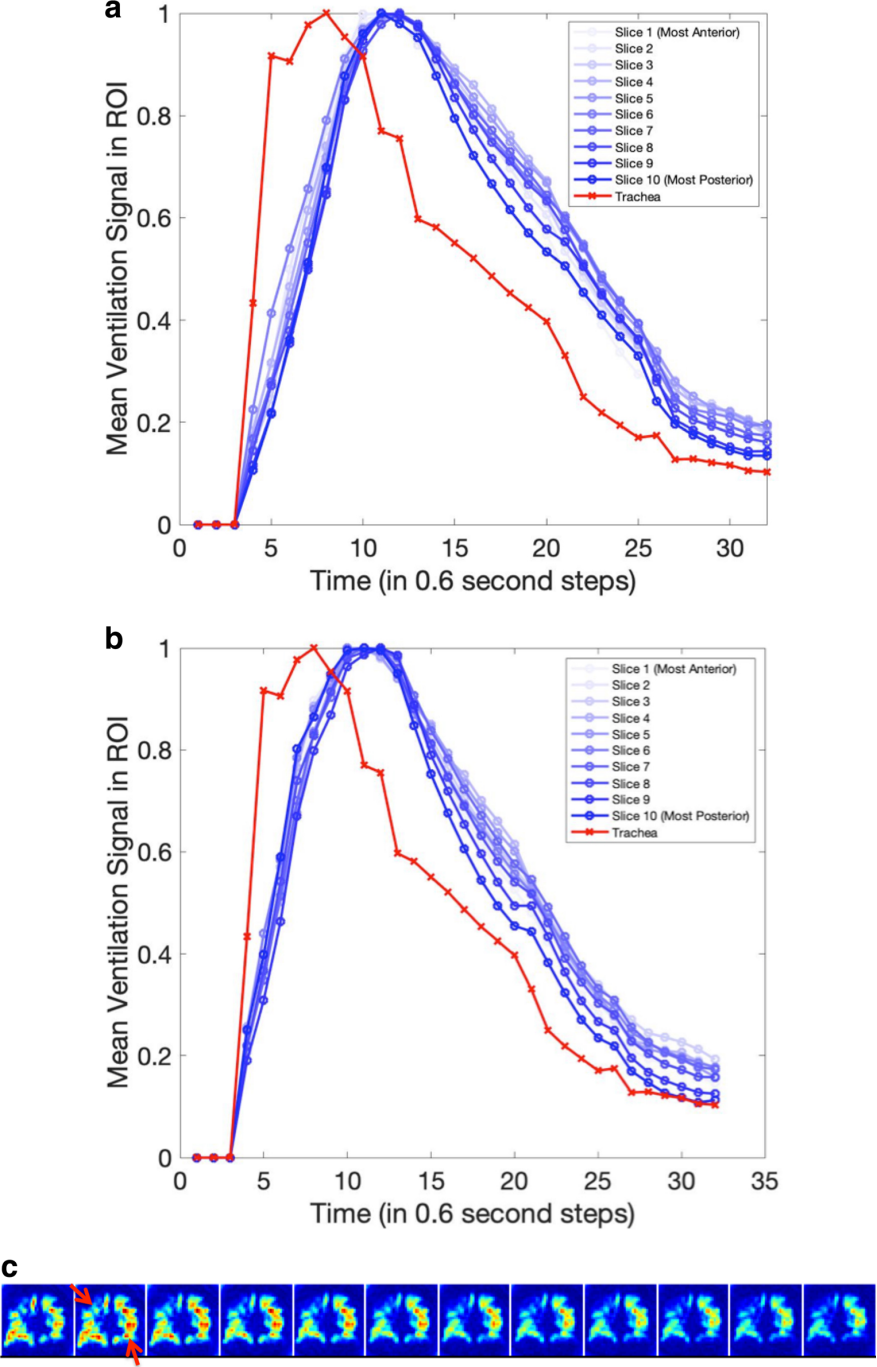
Comparison of the temporal single from the trachea with that from the lung ROIs on various coronal slices in COPD Patient 14. **a** Left lower lung. **b** Right upper lung. **c** Truncated DVeX-MRI sequence demonstrating delayed ventilation in the left lower and right upper lungs. Note the absence of a clear pattern of variation between the coronal slices

Figure 5 shows the temporal ventilation signals in the trachea and lung ROIs for a control participant, a COPD patient with no detectable delayed ventilation (Patient 6), and a COPD patient with delayed ventilation in the right upper lung (Patient 3). The presence of delayed ventilation is clearly demonstrated in the second COPD participant. Figure 6 shows the anterior to posterior coronal temporal signals of a COPD patient with delayed ventilation in both lungs (Patient 14). The trachea signal presented in this case is the average signal computed from several coronal slices, as in the cases presented in Fig. 5. This is because due to tracheal anatomy, there is only signal on the central slices, precluding a coronal slice by slice comparison with the coronal lung ROI signals. There was no clear discrepancy between coronal slices in capturing the delayed ventilation. The corresponding DVeX-MRI images are given in Figs. 5 and 6, truncated for the purpose of illustrating key features in the dynamic sequence.

## Discussion

The utility of DXeV-MRI for assessing delayed ventilation is supported by our experimental findings, where a quantifiable delayed ventilation was observed in at least one lung ROI in 87% of the COPD cohort and none in the control group (*p* = 0.0173), although the difference in group sizes should be noted when interpreting this result. The time delay was measured in the order of seconds with respect to the trachea signal, and the amount of delayed ventilation was quantified using signal-time product difference. Amongst the ROIs where delayed ventilation is detected, the mean time delay was 2.0 seconds (95% CI [1.3, 2.7]), and the mean signal-time product difference was 0.93 (95% CI [0.72, 1.14]). These results demonstrate a level of consistency of the degree of delayed ventilation in cases where it is observed. All cases where delayed ventilation was observed were also confirmed visually on the DXeV images by a clinical radiologist observer.

The prevalence of observed delayed ventilation is consistent with that published in earlier literature using hyperpolarised 3He time-resolved breath-hold MRI [14]. Our findings have established statistically significant correlations between the QCT-derived %LAA and the trachea-ROI covariance, and time delay, computed on a regional basis. This provided evidence that the presence and extent of delayed ventilation measured using our method correlated with the degree of emphysema, as measured on QCT. The ability to assess delayed ventilation may enable the assessment of collateral ventilation, pending further research work and clinical validation, to guide lung volume reduction therapy (LVRT) for certain COPD patients, achieved either surgically by lung resection or bronchoscopically by placing an endobronchial valve. Pulmonary lobes that are most likely to benefit from LVRT are those least affected by collateral ventilation [36]. The short table time of our HP ^129^Xe-MRI scan, in most cases under 5 minutes, would support its use in a wide range of applications without adding substantially to the existing clinical workload.

There are several limitations to our study. First, we have not compared our results to those from Chartis®, the current gold standard method of measuring collateral ventilation. It would be desirable as future work if we make this comparison to further validate our findings.

In this study, adjustment for the variation in the local oxygen concentration was not included in the analysis. T1 may vary between 26 to 33 seconds between different ROIs of the same participant, as we discussed in our previous work [28]. Considering the time delay was calculated over a time period of around 10 seconds, this T1 variation might result in up to 10% variation in signal-time product difference and/or time delay.

Finally, manual delineation of the lung ROIs was performed based on anatomical knowledge of the pulmonary lobes and observed fissures. On coronal DXeV slices, especially where there is a heavy presence of ventilation defects, lobar fissural identification may be challenging, in which case this information had to be extrapolated from neighbouring slices where the fissure was more visible. For this reason, the ROIs were labelled as lung areas, rather than actual pulmonary lobes. To perform a full lobar analysis, visualisation of the horizontal fissures on DXeV would be required, which, from our experience, is not possible on most DVeX-MRI scans involving COPD patients. In our past work with static ^129^Xe-MRI, lobar analysis has been achieved through the use of advanced machine learning tools to co-register MRI data to QCT, the latter of which is far superior in showing the pulmonary fissures. In that work, ^1^H-MRI was used as an intermediary, which provided key MRI anatomical information to facilitate inter-modal registration. However, this was not feasible in this dynamic sequence study, as a separate ^1^H-MRI would be needed for each time point in the breathing cycle. One possible solution to this could involve ventilation map modelling using CT and spirometry test data.

In summary, this study has demonstrated the use of DXeV-MRI as a non-invasive way of detecting and quantifying delayed ventilation in patients with COPD, and providing physiological information on regional pulmonary function during a full breathing cycle.

## Electronic supplementary material

ESM 1 (MP4 390 kb)

ESM 2 (DOCX 173 kb)

## Abbreviations

%LAA: Percentage of low attenuations areas on CT in emphysema
^129^Xe: Xenon isotope 129
^133^Xe: Xenon isotope 133
^3^He: Helium isotope 3
CI: Confidence interval
COPD: Chronic obstructive pulmonary disease
CT: Computed tomography
DV: Delayed ventilation
DXeV: Dynamic hyperpolarised xenon-129 ventilation sequence
FEV1: Forced expiratory volume in one second
FRC: Functional residual capacity
FVC: Forced vital capacity
LVRT: Lung volume reduction therapy
MRI: Magnetic resonance imaging
PTK: Pulmonary Toolkit
QCT: Quantitative computed tomography
ROI: Region of interest
SEOP: Spin-exchange optical pumping
