# Supplementary material for: Delayed ventilation assessment using fast dynamic hyperpolarised Xenon-129 magnetic resonance imaging
Source: Eur Radiol. 2019 Sep 4;30(2):1145–55. doi: 10.1007/s00330-019-06415-1 (PMC6957546; doi:10.1007/s00330-019-06415-1)
Supplement: Supplementary file 2 — (DOCX 173 kb) [file 330_2019_6415_MOESM2_ESM.docx]

**SUPPLEMENTARY MATERIALS**

*
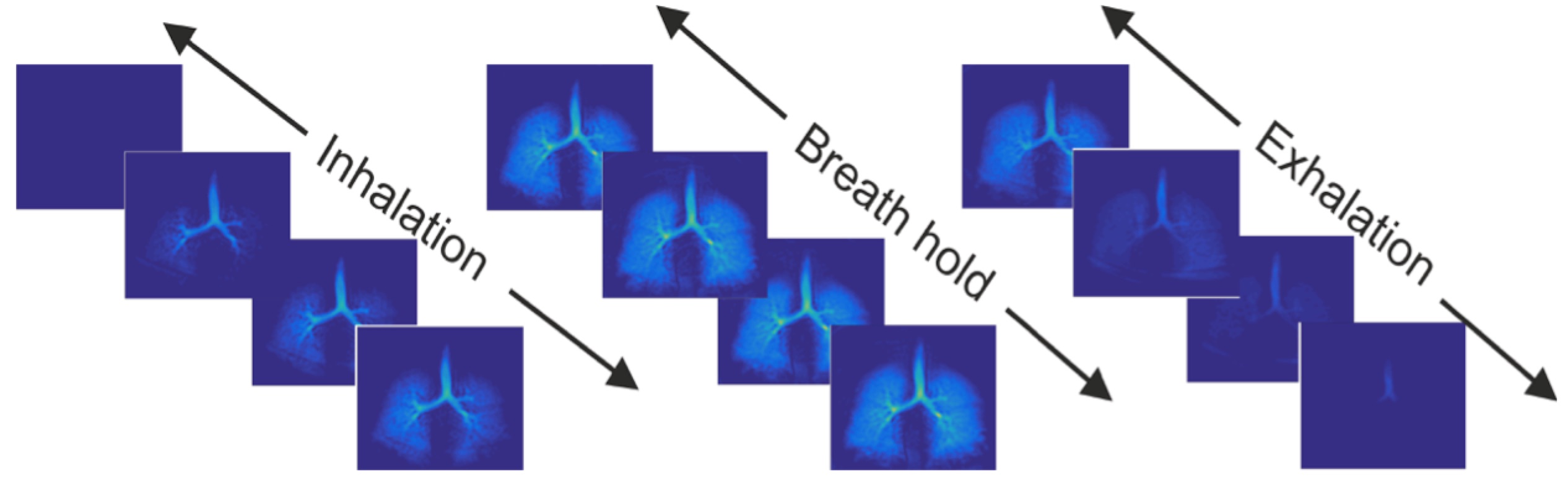
Supplementary Figure: Standard breathing instruction during DXeV-MRI (3 seconds inhalation, 5 seconds breath hold and 3 seconds exhalation), illustrated using projection images acquired from a healthy volunteer.*

*Supplementary Clip: DXeV-MRI demonstrated as a movie clip, from a healthy volunteer*

***Study Recruitment Criteria***

**Control Cohort**

***Inclusion Criteria***

1. Male, aged > 18 years.
2. Patients with previous histologically verified testicular germ cell tumour who have successfully undergone resection and do not and have never had metastatic disease.
3. Normal chest CT, confirmed subjectively by a consultant thoracic radiologist, and no evidence of emphysema on CT density mapping to have been performed no more than one year prior to enrolment.
4. Normal spirometry indices (>80% predicted FEV1 for age and height) and normal arterial oxygen saturations (SaO2), normal carbon monoxide transfer factor, and generally in good health with no subjective exercise limitation.
5. Current non-smokers with no significant smoking history (≤10 pack years) and no history of respiratory disease.
6. WHO performance status 0.

Criterion 2 is used to justify the radiation dose involved in the chest CT, as per IRMER regulation, where it is required as part of the standard clinical care of those participants.

***Exclusion Criteria***

1. Patients with a history of nodal or metastatic germ cell tumour.
2. Patients with a prior history of chemotherapy or radiotherapy at study entry.
3. Prior history of thoracic surgery or significant chest trauma
4. Prior history of significant smoking or respiratory disease.
5. The presence of another malignancy, where the extent of disease or treatment for that condition may interfere with the study endpoints.
6. Any psychological, familial, sociological or geographical condition potentially hampering compliance with the study protocol and follow-up schedule.
7. Inability to lie flat for imaging.
8. Contra-indications to receiving iodine-based contrast during thoracic CT – those with marked renal failure not on dialysis, known allergy to contrast medium, history of anaphylaxis, known or suspected thyroid carcinoma and inability to gain intra-venous access.
9. Contraindications to MRI examination including indwelling pacemaker, incompatible metallic implant, severe claustrophobia, history of metallic intra-ocular foreign body.
10. Epilepsy requiring on-going medical treatment, or a seizure within the past year.

**Study Cohort**

***Inclusion Criteria***

1. At least mild disease (stage II-IV on GOLD criteria classification, forced expiratory volume in 1 second (FEV1)<80% predicted and FEV1/forced vital capacity (FVC) <70%)
2. Significant smoking history (>15 pack years) or other definite cause of COPD
3. Over the age of 18 and able to give informed consent.

***Exclusion Criteria***

1. The presence of co-existent cardio-pulmonary disease that predominated over COPD and might confound result interpretation (e.g. asthma, bronchiectasis, cystic fibrosis, lung cancer, uncontrolled heart failure, frequent unstable angina, respiratory muscle weakness)
2. Any psychological, familial, sociological or geographical condition potentially hampering compliance with the study protocol and follow-up schedule.
3. Inability to lie flat for imaging.
4. Contra-indications to receiving iodine-based contrast during thoracic CT – those with marked renal failure not on dialysis, known allergy to contrast medium, history of anaphylaxis, known or suspected thyroid carcinoma and inability to gain intra-venous access.
5. Contraindications to MRI examination including indwelling pacemaker, incompatible metallic implant, severe claustrophobia, history of metallic intra-ocular foreign body.
6. Epilepsy requiring on-going medical treatment, or a seizure within the past year.
